# Changing times: The impact of gram-negative breakpoint changes over the previous decade

**DOI:** 10.1017/ash.2022.301

**Published:** 2022-10-07

**Authors:** Wes M. Johnson, Justin A. Clark, Katherine Olney, Donna R. Burgess, David S. Burgess

**Affiliations:** 1 University of Kentucky Chandler Medical Center, Lexington, Kentucky; 2 University of Kentucky College of Pharmacy, Lexington, Kentucky

## Abstract

We assessed breakpoint changes of 13,101 Enterobacterales and *Pseudomonas aeruginosa* isolates from the past decade. All β-lactams and fluoroquinolones demonstrated decreased susceptibilities following breakpoint changes. *Enterobacter cloacae* experienced the largest average decrease in susceptibility amongst the Enterobacterales at 5.3% and *P. aeruginosa* experienced an average decrease in susceptibility of 9.3%.

The Clinical and Laboratory Standards Institute (CLSI) is a governing body responsible for establishing minimum inhibitory concentration (MIC) breakpoints. Over the past decade, multiple changes to MIC breakpoints have occurred for both Enterobacterales and *Pseudomonas aeruginosa*. As noted by the CLSI subcommittee on antimicrobial susceptibility testing, a microorganism’s susceptibility to an antimicrobial agent may decrease over time, thus warranting the refinement of MIC breakpoints.^1^ Additionally, the new breakpoints were made to better fit MIC distribution patterns emerging in clinical practice.^2^

Individual breakpoint changes can be found both on the CLSI website^3^ and summarized by Humphries et al.^4^ These changes made by CLSI directly affect the susceptibility of antibiotics. Specifically, susceptibilities of β-lactams and fluoroquinolones are of particular concern due to their common use within the inpatient setting.^5^

Questions remain regarding the quantitative effect of breakpoint changes on institutional susceptibilities. One study demonstrated a decrease in ciprofloxacin susceptibilities of 5% for Enterobacterales^6^ and another demonstrated increases in institutional resistance of 6% for *Escherichia coli* and *Enterobacter cloacae.* ^7^ As bacteria continue to adapt, we must update breakpoints accordingly, otherwise we risk the provision of suboptimal treatment. In this study, we have described the impact of CLSI breakpoint changes on gram-negative organism susceptibilities in the last decade at our academic medical center.

## Methods

This single-center retrospective study was conducted at the University of Kentucky Chandler Medical Center. We collected consecutive nonduplicate clinical isolates of *Enterobacter cloacae*, *Escherichia coli*, *Klebsiella aerogenes*, *K. oxytoca*, *K. pneumoniae*, and *Pseudomonas aeruginosa* between the years 2010 and 2019. We assessed aztreonam, cefepime, ceftazidime, ceftriaxone, ertapenem, and meropenem for the Enterobacterales. Fluoroquinolones were not evaluated because automated panels were unable to determine low enough MICs. We assessed ciprofloxacin, levofloxacin, meropenem and piperacillin-tazobactam for *P. aeruginosa*. The institutional isolates and their respective MICs were obtained from the institutional antibiogram data set. This data set also stratifies isolates that come from specific intensive care units (ICUs) including the cardiovascular ICU (CVICU), the medical ICU (MICU), and the neurosurgical ICU (NSICU).

Susceptibilities were assessed based upon historic CLSI breakpoints in 2010 as well as the current CLSI breakpoints from the most recent CLSI MIC breakpoints, the 29th edition of document M100, published in 2019. MICs were determined using automated panels in BD Phoenix software (Becton-Dickinson, Franklin Lakes, MJ). The genus and species of isolates were determined through use of matrix-assisted laser desorption ionization time-of-flight mass spectrometry (MALDI-TOF). Additionally, if further susceptibility testing was warranted, then an E-test was performed.

Susceptibility differences were calculated by subtracting the new susceptibilities from the old susceptibilities. Statistical analysis was performed utilizing the McNemar test to compare previous susceptibilities to current susceptibilities. *P <* 0.05, was considered statistically significant.

## Results

In total, 13,101 clinical isolates were collected over the 10-year period. Of these isolates, 10,003 were Enterobacterales isolates and 3,098 were *P. aeruginosa* isolates. The Enterobacterales group consisted of 5,273 *E. coli*, 1,237. *E. cloacae*, 471 *K. aerogenes*, 610 *K. oxytoca*, and 2,412 *K. pneumoniae* isolates. Table 1 describes the susceptibility changes for Enterobacterales at Chandler Hospital and the ICUs. At Chandler Hospital, *E. cloacae* experienced the largest average change in susceptibility for all antibiotics at 5.3%, followed by *K. aerogenes* at 3.3%, *E. coli* at 2.1%, *K. pneumoniae* at 1.9%, and *K. oxytoca* at 1.8%. Table 1 also demonstrates that each organism at Chandler Hospital has at least 1 antibiotic with a statistically significant decrease in susceptibility, with *E. coli* and *E. cloacae* experiencing statistically significant changes for all antibiotics. Table 2 describes the susceptibility changes for *P. aeruginosa.* At Chandler Hospital, there was an average change in susceptibility of 9.3% for *P. aeruginosa*, with piperacillin-tazobactam demonstrating a 15.4% decrease in susceptibility. All drugs assessed with *P. aeruginosa* had a statistically significant reduction in susceptibilities (Table 2). Results from the ICU follow a similar trend as Chandler Hospital, but with a smaller number of isolates (Tables 1 and 2).

**Table 1.** Changes in Rates of Susceptibility by Organism for Enterobacterales. Changes in susceptibilities are listed for Chandler Hospital, cardiovascular ICU, medical IDU, and the neurosurgical ICU. The individual changes are listed, along with the average. ATM aztreonam, CAZ ceftazidime, CEF cefepime, CRO ceftriaxone, ETP Ertapenem, MEM meropenemtablet^a,b^

Chandler Hospital (All Units)															
	*E. coli* n = 5,273			*E. cloacae* n = 1,237			*K. aerogenes* n = 471			*K. oxytoca* n = 610			*K. pneumoniae* n = 2,412		
Agent	Old (%)	New (%)	p-value	Old (%)	New (%)	p-value	Old (%)	New (%)	p-value	Old (%)	New (%)	p-value	Old (%)	New (%)	p-value
**ATM**	92.5	89.0	<0.01	73.4	71.8	<0.01	76.4	75.4	0.06	95.7	95.1	0.13	94.4	94.2	0.06
**CEF**	93.6	90.7	<0.01	94.7	88.1	<0.01	98.5	95.7	<0.01	99.8	99.0	0.06	96.2	94.7	<0.01
**CAZ**	93.8	92.2	<0.01	75.1	74.3	<0.01	73.7	72.4	0.03	98.5	98.4	1	93.4	92.3	<0.01
**CRO**	79.4	75.4	<0.01	58.1	51.7	<0.01	58.5	50.2	<0.01	94.6	86.5	<0.01	85.9	79.9	<0.01
**ETP**	99.9	99.6	<0.01	97.8	82.9	<0.01	98.7	93.5	<0.01	100	99.3	0.13	99.2	97.0	<0.01
**MEM**	99.9	99.8	<0.01	98.5	97.2	<0.01	99.4	98.3	0.06	100	99.8	1	99.1	98.5	<0.01
**Average Δ**	2.1%			5.3%			3.3%			1.8%			1.9%		

Cardiovascular ICU															
	*E. coli* n = 283			*E. cloacae* n = 78			*K. aerogenes* n = 53			*K. oxytoca* n = 48			*K. pneumoniae* n = 215		
Agent	Old (%)	New (%)	p-value	Old (%)	New (%)	p-value	Old (%)	New (%)	p-value	Old (%)	New (%)	p-value	Old (%)	New (%)	p-value
**ATM**	91.0	88.5	0.02	75.6	73.1	0.5	73.6	73.6	1	93.8	93.8	1	92.1	92.1	1
**CEF**	91.1	87.9	<0.01	94.8	87.0	0.03	98.1	94.3	0.5	100	97.9	1	93.9	92.5	0.25
**CAZ**	92.5	90.0	0.02	72.7	70.1	0.5	71.2	67.3	0.5	93.8	93.8	1	91.6	90.2	0.25
**CRO**	79.2	77.6	0.25	57.4	53.7	0.5	53.1	50.0	1	90.9	90.9	1	87.8	84.5	0.06
**ETP**	99.6	99.6	1	97.4	80.5	<0.01	98.1	92.3	0.25	100	100	1	100	96.3	<0.01
**MEM**	100	99.3	0.5	94.9	94.9	1	98.1	96.2	1	100	100	1	100	98.6	0.25
**Average Δ**	1.8%			5.6%			3.1%			0.4%			1.9%		

Medical ICU															
	*E. coli* n = 330			*E. cloacae* n = 73			*K. aerogenes* n = 27			*K. oxytoca* n = 26			*K. pneumoniae* n = 185		
Agent	Old (%)	New (%)	p-value	Old (%)	New (%)	p-value	Old (%)	New (%)	p-value	Old (%)	New (%)	p-value	Old (%)	New (%)	p-value
**ATM**	87.2	85.0	0.02	66.7	66.7	1	60.0	58.6	1	96.2	96.2	1	81.0	80.4	1
**CEF**	89.6	84.4	<0.01	96.3	92.6	<0.01	88.7	77.5	1	100	100	1	89.2	85.4	0.02
**CAZ**	91.2	88.7	<0.01	65.4	65.4	1	61.6	60.3	1	100	100	1	79.8	76.5	0.03
**CRO**	68.2	65.4	0.06	60.0	60.0	1	43.1	43.1	1	100	95	1	61.9	58.1	0.13
**ETP**	100	99.7	1	100	91.7	<0.01	93.1	66.7	0.5	100	100	1	96.2	86.1	<0.01
**MEM**	100	99.7	1	96.3	96.3	0.5	94.4	91.7	1	100	100	1	95.7	93.5	0.13
**Average Δ**	2.2%			2%			7.2%			0.8%			4%		

Neurosurgical ICU															
	*E. coli* n = 290			*E. cloacae* n = 66			*K. aerogenes* n = 35			*K. oxytoca* n = 40			*K. pneumoniae* n = 146		
Agent	Old (%)	New (%)	p-value	Old (%)	New (%)	p-value	Old (%)	New (%)	p-value	Old (%)	New (%)	p-value	Old (%)	New (%)	p-value
**ATM**	92.1	91.4	0.5	82.9	80.0	1	75.0	75.0	1	97.5	97.5	1	92.7	92.7	1
**CEF**	93.1	91.7	0.13	100	100	0.03	95.3	85.9	1	100	100	1	96.7	96.0	1
**CAZ**	93.1	93.1	1	82.9	82.9	1	75.8	75.8	1	100	100	1	92.1	92.1	1
**CRO**	86.4	83.5	0.06	73.9	69.6	0.25	66.0	59.6	1	100	94.4	1	86.4	84.0	0.5
**ETP**	100	100	1	97.1	91.2	<0.01	98.4	78.1	0.5	100	100	1	98.0	96.0	0.25
**MEM**	100	100	1	100	100	1	98.5	98.5	1	100	100	1	98.7	97.4	0.5
**Average Δ**	0.8%			2.2%			6%			0.9%			1.1%		

Note. ATM, aztreonam; CAZ, ceftazidime; CEF, cefepime; CRO, ceftriaxone; ETP, ertapenem; MEM, meropenem; ICU, intensive care unit.

a Changes in susceptibilities are listed for Chandler Hospital, cardiovascular ICU, medical IDU, and the neurosurgical ICU.

b The individual changes are listed, along with the average.

**Table 2.** Changes in Rates of Susceptibility by Organism for P. aeruginosa. Changes in susceptibilities are listed for Chandler Hospital, cardiovascular ICU, medical IDU, and the neurosurgical ICU. The individual changes are listed, along with the average. ABX antibiotic, CPX ciprofloxacin, LVX levofloxacin, MEM meropenem, TZP piperacillin-tazobactam^a,b^

Chandler Hospital (All Units)			
*P. aeruginosa* (n = 3,098)			
Agent	Old (%)	New (%)	p-value
**CPX**	67.5	58.7	<0.01
**LVX**	63.2	55.6	<0.01
**MEM**	78.0	72.7	<0.01
**TZP**	88.1	72.7	<0.01
**Average Δ**	9.3%		
Cardiovascular ICU			
***P. aeruginosa*** **(n = 265)**			
Agent	**Old (%)**	**New (%)**	**p-value**
**CPX**	71.7	63.9	<0.01
**LVX**	58.5	49.2	<0.01
**MEM**	74.2	67.4	<0.01
**TZP**	83.1	65.0	<0.01
**Average Δ**	10.5%		
Medical ICU			
***P. aeruginosa*** **(n = 422)**			
Agent	**Old (%)**	**New (%)**	**p-value**
**CPX**	47.5	39.8	<0.01
**LVX**	42.0	32.9	<0.01
**MEM**	59.8	53.8	<0.01
**TZP**	80.7	55.8	<0.01
**Average Δ**	11.9%		
Neurosurgical ICU			
***P. aeruginosa*** **(n = 146)**			
Agent	**Old (%)**	**New (%)**	**p-value**
**CPX**	74.3	65.0	<0.01
**LVX**	70.1	63.9	<0.01
**MEM**	78.1	73.3	0.02
**TZP**	89.5	73.4	<0.01
**Average Δ**	9.1%		

Note. ABX, antibiotic; CPX, ciprofloxacin; LVX, levofloxacin; MEM, meropenem; TZP piperacillin-tazobactam; ICU, intensive care unit.

a Changes in susceptibilities are listed for Chandler Hospital, cardiovascular ICU, medical ICU, and the neurosurgical ICU.

b The individual changes are listed, along with the average.

## Discussion

In this study, we have demonstrated decreased institutional susceptibilities for gram-negative pathogens following the implementation of updated CLSI breakpoints. In this study, both Enterobacterales and *P. aeruginosa* susceptibilities decreased institution-wide and in the various ICUs.

Our findings may not align with other institutions due to differing local susceptibilities. Shealy et al^6^ assessed the effects of the MIC breakpoint changes to fluoroquinolone susceptibilities. Their findings demonstrated a decrease in susceptibility of levofloxacin to *P. aeruginosa* by 8% after the implementation of updated MIC breakpoint, which is in line with our findings. However, Shealy et al^6^ reported more favorable susceptibilities for levofloxacin, with 89% of *P. aeruginosa* isolates susceptible initially. Within our institution, this rate was only 63%.

The decreased susceptibilities make it clear that microbiology laboratories should accept new MIC breakpoints. However, one study assessed the uptake of current CLSI breakpoints by microbiology laboratories in California and found that ∼1 in 3 labs were utilizing out-of-date breakpoints.^8^ This finding may stem from the fact that many laboratories will take at least a year to implement new breakpoints.^4^ This delay in the uptake of breakpoint changes stems from antimicrobial susceptibility testing manufacturers being required to follow the breakpoints set by the Food and Drug Administration (FDA), who will accept CLSI breakpoint recommendations after an extensive review process.^4^

Although our study has demonstrated decreased susceptibilities, there is no correlation with clinical outcomes. Another study assessed clinical outcomes by determining mortality associated with previous and current levofloxacin breakpoints with *Enterobacterales*. The outdated levofloxacin MIC was a predictor of 30-day mortality, with an odds ratio of 6.05. Additionally, the outdated MIC was associated with the emergence of resistance, 25% versus 7.5%.^9^

In conclusion, changes in breakpoints had a significant impact on the susceptibility of all antimicrobials for *P. aeruginosa* at our institution, both hospital-wide and in intensive care units. Although the impact was less for Enterobacterales isolates, ertapenem, ceftriaxone, and cefepime demonstrated significant susceptibility changes. Understanding and evaluating the impact of the breakpoint changes is of paramount importance. Institutions should ensure that their breakpoints are up to date to allow for the most optimized treatment.
